# A Novel Supercritical CO_2_ Foam System Stabilized With a Mixture of Zwitterionic Surfactant and Silica Nanoparticles for Enhanced Oil Recovery

**DOI:** 10.3389/fchem.2019.00718

**Published:** 2019-10-29

**Authors:** Weitao Li, Falin Wei, Chunming Xiong, Jian Ouyang, Liming Shao, Mingli Dai, Pingde Liu, Dongxing Du

**Affiliations:** ^1^Department of Oilfield Chemicals, Research Institute of Petroleum Exploration & Development, PetroChina, Beijing, China; ^2^College of Electromechanical Engineering, Qingdao University of Science and Technology, Qingdao, China

**Keywords:** supercritical CO_2_ foam, silica nanoparticles, foam stability, rheological properties, mobility control

## Abstract

In order to improve the CO_2_ foam stability at high temperature and salinity, hydrophilic silica nanoparticles (NPs) were added into a dilute zwitterionic surfactant solution to stabilize supercritical CO_2_ (SC-CO_2_) foam. In the present paper, the foaming capacity and stability of SC-CO_2_ foam were investigated as a function of NP concentration at elevated temperatures and pressures. It was observed that the drainage rate of SC-CO_2_ foam was initially fast and then became slower with NPs adsorption at the gas-liquid interface. The improved foam stability at high temperature was attributed to the enhanced disjoining pressure with addition of NPs. Furthermore, an obvious increase in the foam stability was noticed with the increasing salinity due to the screening of NP charges at the interface. The rheological characteristics including apparent viscosity and surface elasticity, resistance factor, and microstructures of SC-CO_2_ foam were also analyzed at high temperature and pressure. With addition of 0.7% NPs, SC-CO_2_ foam was stabilized with apparent viscosity increased up to 80 mPa·s and resistance factor up to 200. Based on the stochastic bubble population (SBP) model, the resistance factor of SC-CO_2_ foam was simulated by considering the foam generation rate and maximum bubble density. The microstructural characteristics of SC-CO_2_ foam were detected by optical microscopy. It was found that the effluent bubble size ranged between 20 and 30 μm and the coalescence rate of SC-CO_2_ foam became slow with the increasing NP concentration. Oscillation measurements revealed that the NPs enhanced surface elasticity between CO_2_ and foam agents for resisting external disturbances, thus resulting in enhanced film stability and excellent rheological properties.

## Introduction

CO_2_ flooding is a widely applied enhanced oil recovery (EOR) method due to its easy miscibility with crude oil, thereby resulting in an expanded oil volume and a decrease in oil viscosity (Sanders et al., 2012; Mukherjee et al., 2014). However, the occurrence of an early CO_2_ breakthrough leads to a poor sweep efficiency and high operating cost due to gravity override, viscous fingering, and formation heterogeneity (Chang and Grigg, 1999; Farajzadeh et al., 2012; Ayesh et al., 2017). CO_2_ foam or chemical gels are the most common agents to improve the macroscopic sweep efficiency by controlling the mobility of CO_2_ in high-permeability zones. Polymer gels are often used to plug thief zones by diverting CO_2_ to other zones (Chang and Grigg, 1999; Zhang et al., 2019); however, they usually exist near the wellbore and manifest a limited effect on deep formation control (Zhang and Seright, 2007). CO_2_ foam, which is a dispersion of discontinuous CO_2_ in the liquid phase, can overcome the aforesaid drawbacks and is regarded as one of the effective mobility control agents for CO_2_ flooding. CO_2_ foam is extensively applied to improve the sweep efficiency of heterogeneous reservoirs by reducing gas relative permeability and increasing gas viscosity (Lotfollahi et al., 2016). When the reservoir temperature and pressure exceed the critical point of CO_2_ (31.1°C and 7.37 MPa, respectively), gaseous CO_2_ is usually transformed into a dense, liquid-like fluid (supercritical CO_2_) (Chang and Grigg, 1999). The viscosity of supercritical CO_2_ generally remains very low, whereas its density (0.5–0.9 g/cm^3^) is found to be significantly higher than that of gaseous CO_2_. Therefore, the physical properties of supercritical CO_2_ and gaseous CO_2_ are completely different under ambient conditions. Moreover, when supercritical CO_2_ is dispersed into a surfactant solution, CO_2_ foam manifests much better solubility, pH, texture, and mobility as compared to gaseous CO_2_ foam.

However, the stability of CO_2_ foam generally deteriorates under harsh reservoir conditions (high temperature and salinity) (Chen et al., 2015; Chang et al., 2018). Foam stability can be considered as the stability of a bubble film and is determined by numerous factors including bulk viscosity, surface viscoelasticity, Marangoni effect, and disjoining pressure (Tamura et al., 1997). In addition, film stability is also affected by the temperature, salinity, pressure, and pore structure of a porous media. In order to improve foam stability, different foaming agents including surfactants (Basheva et al., 1999; Dhanuka et al., 2006), nanoparticles (NPs) (Basheva et al., 1999; Stephanie et al., 2008; Worthen et al., 2014), and polymers (Basheva et al., 1999; Petkova et al., 2012; Kalyanaraman et al., 2016) have been incorporated into CO_2_ foam. The apparent viscosity of SC-CO_2_ foam stabilized with surfactants and polymers is significantly higher (by several orders of magnitude) than that of a pure gaseous foam (Da et al., 2018). However, the strength or solubility of surfactants and polymers starts to degrade under high temperatures and salinity, thereby resulting in poor foam stability. NPs are the most effective agent to stabilize CO_2_ foam due to their high chemical stability at high temperature and salinity (Alzobaidi et al., 2017). Silica nanoparticles can form compact coherent particle shells at the gas-liquid interface, thus resulting in improved foam stability by resisting film deformation (Horozov, 2008). The effectiveness of solid particles depends on their sizes, shapes, concentrations, and wettability. When wetted (θ = 0°) or dewetted (θ = 180°) solid particles remain dispersed in the liquid phase or the gas phase, no stable liquid film is generally formed. When the contact angle (θ) ranges between 0° and 180°, solid particles start to agglomerate in liquid films. The aqueous and gaseous phases are the preferred accumulation sites for hydrophilic (0° < θ < 90°) and hydrophobic particles (90° < θ < 180°), respectively (Aveyard and Clint, 1995).

It is found that hexadecyl hydroxypropyl sulfobetaine (HHSB; a zwitterionic surfactant) can generate SC-CO_2_ foam under high temperature (up to 90°C) and salinity (25 × 10^4^ mg/L). However, as temperature was higher than 90°C, the stability of SC-CO_2_ foam declined sharply. In the present work, in order to improve the stability of SC-CO_2_ foam at high temperatures, hydrophilic silica NPs were added to a dilute HHSB solution. Furthermore, previous researches have mainly focused on the capacity of NPs-stabilized CO_2_ foam at atmospheric pressure and room temperature. Thus, we presented a detailed investigation on effect of NPs on SC-CO_2_ foam. The stability, rheological characteristics and stabilization mechanism of SC-CO_2_ foam were explored with an increase in the NP concentration.

## Materials and Methods

### Materials

HHSB (C_16_H_33_N(CH_3_)_2_CH_2_CH (OH)CH_2_SO_3_) was procured from Shanghai Nuosong Chemical Co. Ltd., China. Hydrophilic silica nanoparticles (average size of 65 nm) were purchased as a 30% concentration aqueous solution from Sigma-Aldrich. Sodium chloride and calcium chloride were obtained from Beijing Chemical Works, China. Four types of the formation brine (the amounts of dissolved solid were 2 × 10^4^ mg/L, 5 × 10^4^ mg/L, 7 × 10^4^ mg/L, and 10 × 10^4^ mg/L) were prepared by adding NaCl and CaCl_2_ (in a ratio of 9:1) into deionized (DI) water.

### Evaluation of SC-CO_2_ Foaming Capacity and Stability

**Figure 1:**
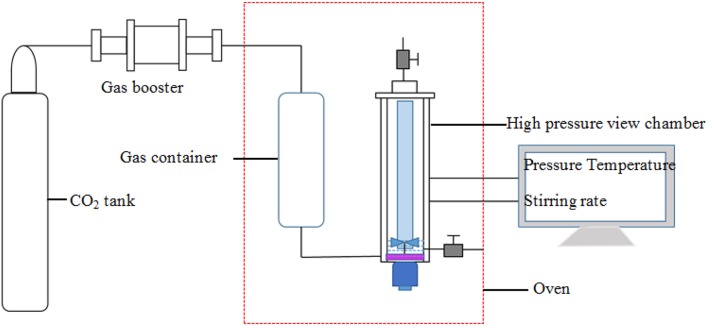
Schematic diagram of the apparatus to evaluate foam stability.

The schematic diagram of the apparatus used for assessing the capacity and stability of SC-CO_2_ foam at high temperatures and pressures is displayed in Figure 1. CO_2_ in the gas tank was first pressurized to the desired pressure and then put into the gas container in the oven. After CO_2_ was heated to the desired temperature, supercritical CO_2_ was injected into the high-pressure view chamber. The HHSB/NPs solution (100 mL) was then pumped into the chamber. Finally, when the desired pressure and temperature were attained in the chamber, the mixture was sheared at 2,000 rpm for 5 min. The maximum foaming volume was recorded to assess the foaming ability of foaming agents. The liquid volume separated from the foam was recorded as a function of time. In order to evaluate the foam stability, the half-life of the foam was defined as the time when half of the liquid was drained from the foam.

### Rheological Measurements

**Figure 2:**
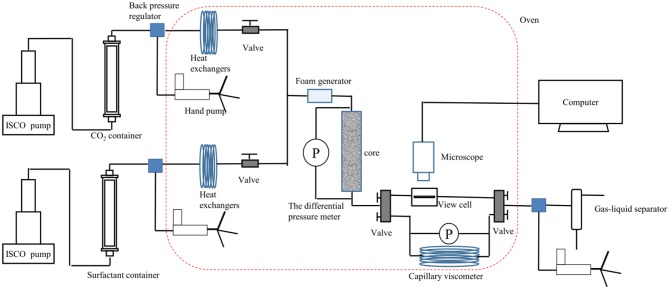
Flow loop apparatus for evaluating SC-CO_2_ foam apparent viscosity, resistance factor and bubble texture at 70°C and 8 MPa.

The apparent viscosity of SC-CO_2_ foam at high temperatures and pressures was measured by a capillary tube viscometer (internal diameter = 4 mm and length = 8,000 mm) (Figure 2). Foaming agents and SC-CO_2_ were coinjected into the capillary tube in a gas-liquid ratio of 2:1. The pressure difference in the capillary tube was recorded as a function of time after SC-CO_2_ foam reached the steady-state. The shear rate and the shear stress at the foam wall were calculated by Equations (1) and (2). As a non-Newtonian fluid, the rheological characteristics of SC-CO_2_ foam was described by the power-law model (Equation 3) (Xiao et al., 2017). The power-law exponent and the consistency coefficient were obtained from the logarithm plot of shear stress vs. shear rate. The shear rate and the apparent viscosity at the SC-CO_2_ foam wall were calculated by Equations 4 and 5.

(1) $$\begin{array}{r} \gamma_{N}=\frac{8v_{f}}{D} \end{array}$$

(2) $$\begin{array}{r} \tau_{N}=\frac{D\Delta P}{4L} \end{array}$$

(3) $$\begin{array}{r} \tau =K\gamma^{n} \end{array}$$

(4) $$\begin{array}{r} \gamma =\gamma_{N}\left(\frac{3n+1}{4n}\right) \end{array}$$

(5) $$\begin{array}{r} \mu_{a}=\frac{\tau }{\gamma }=K\gamma^{n-1} \end{array}$$

where *v*_*f*_ is the velocity of SC-CO_2_ foam in the capillary, *D* is the inner diameter of the capillary tube, Δ*P* is the pressure difference between two capillary ends, *L* is the length of the capillary tube, *n* and *k* are the power-law exponent and the consistency coefficient, respectively, and μ_*a*_ is the apparent viscosity of SC-CO_2_ foam.

### Steady-State SC-CO_2_ Foam Test Under HTHP Conditions

The foaming capacity and the microstructures of SC-CO_2_ foam were evaluated by the core flooding experiment, and the schematic diagram of the flow loop apparatus is exhibited in Figure 2. The permeability and porosity of the foam were first determined. Foaming agents and CO_2_ were coinjected into the core in a gas-liquid ratio of 2:1 until the foam flow reached the steady-state. The steady-state foam was then injected in the high-pressure view cell (path length of 100 μm). The foam flow graphs were obtained by a Leica S6D camera. The parameters of the cores and foaming agent compositions are presented in Table 1. The resistance factor was calculated to characterize the foam strength, and the bubble size was detected by ImageJ software.

**Table 1:** Summary of core flooding tests.

**Run no**.	**Length/cm**	**Cross-sectional area/cm^**2**^**	**Pore volume/cm^**2**^**	**Permeability/mD**	**Chemical formula**
1	9.95	4.79	10.21	657.7	0.05% HHSB
2	9.90	4.79	10.34	654.5	0.05% HHSB + 0.3% NPs
3	9.78	4.79	10.34	646.8	0.05% HHSB + 0.5% NPs
4	9.51	4.79	9.9	628.6	0.05% HHSB + 0.7% NPs

The resistance factor was calculated through the following equation:

(6) $$\begin{array}{r} RF{=}^{\frac{k_{w}}{\mu_{w}}}{╱}_{\frac{k_{f}}{\mu_{f}}}=\frac{P_{f}}{P_{0}} \end{array}$$

Where *RF* is the resistance factor, *k*_*w*_ is the effective permeability of water, μ_*w*_ is water viscosity, *k*_*f*_ is the effective permeability of foam, μ_*f*_ is foam viscosity, *P*_*f*_ is the pressure difference produced by SC-CO_2_ foam, and *P*_0_ is the pressure difference produced by formation water.

### Measurement of Surface Rheological Properties Under HTHP Conditions

The dilational surface elasticity between CO_2_ and surfactant solutions were measured by an interfacial tensiometer (Rame-hart instrument, France) based on the oscillating drop method). The surface elasticity (E) of SC-CO_2_ foam was calculated as:

(7) $$\begin{array}{r} E=\frac{d\gamma }{d\;ln\;A} \end{array}$$

The surface dilatational viscosity (η_d_) of SC-CO_2_ foam was determined as:

(8) $$\begin{array}{r} \Delta \gamma =\eta_{d}\frac{d\;ln\;A}{dt} \end{array}$$

The surface dilatational modulus of SC-CO_2_ foam was calculated as:

(9) $$\begin{array}{r} E^{*}=E^{\prime }+iE^{\prime \prime } \end{array}$$

where γ denotes surface tension, *A* is the surface area of SC-CO_2_ foam, Δγ is the surface tension difference between a constantly (logarithmically) expanding surface and the equilibrium surface, *E*′ is the storage modulus, and *E*″ is the loss modulus.

## Results and Discussion

### Effects of Silica NPs on the Foaming Capacity of SC-CO_2_ Foam

**Figure 3:**
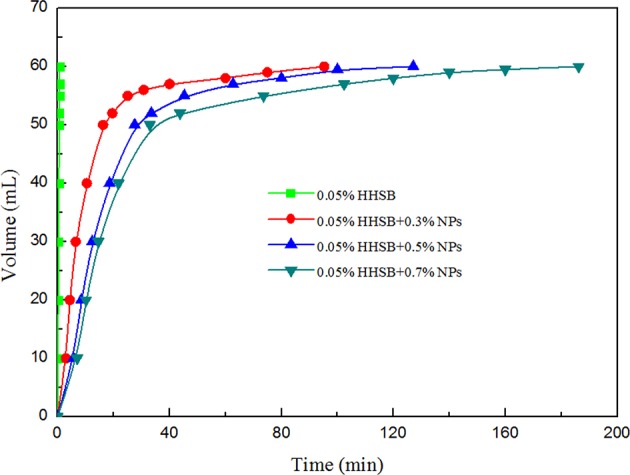
Change in the drainage volume as a function of time at 70°C and 8 MPa for different nanoparticle concentration at the HHSB concentration of 0.05%.

**Figure 4:**
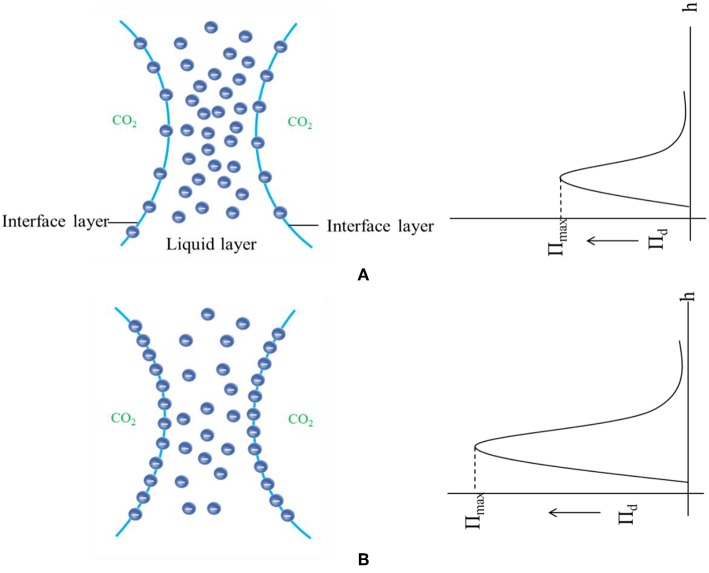
Schematic of disjoining pressure between foam films as a function of film thickness before **(A)** and after **(B)** nanoparticle adsorption at the gas-liquid interface.

**Figure 5:**
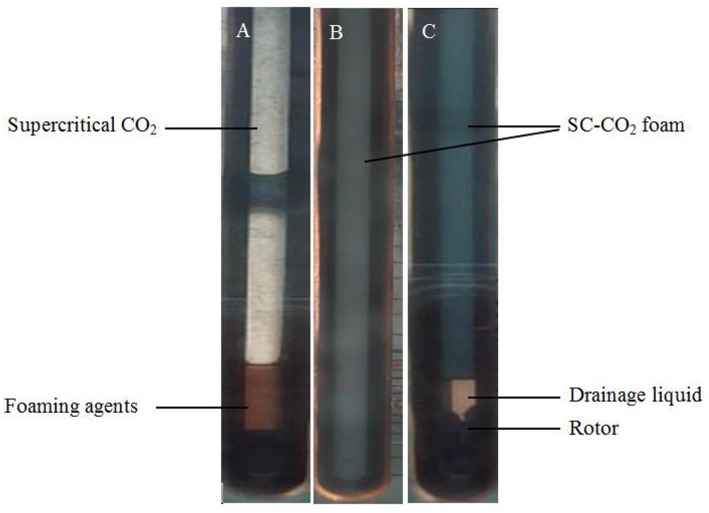
The Foaming process of SC-CO_2_ foams stabilized with 0.05% HHSB and 0.5% NPs at 8 MPa and 70°C. **(A)** The foaming agents before shearing, **(B)** SC-CO_2_ foam immediately after shearing was stopped, and **(C)** the drainage process after shearing was stopped.

When SC-CO_2_ foam was formed in the HPHT cell under the mixing of CO_2_ and foaming agents, the stability of SC-CO_2_ foam was mainly controlled by the gravitational drainage of lamellae, thus resulting in film thinning and the rupture of foam bubbles (Heller and Kuntamukkula, 1987). The drainage rate was used to characterize the stability of SC-CO_2_ foam. The evolution of drainage volume with time is displayed in Figure 3. The drainage rate of SC-CO_2_ foam without NPs was very fast, and the drainage volume reached 60 mL in less than a min. However, the drainage rate declined rapidly after the addition of NPs. It was also noticed that when the drainage liquid volume was <50 mL, a relatively fast drainage rate was achieved. However, when the drainage liquid volume was >50 mL, the drainage rate became very slow. Table 2 summarized the change of drainage rare as a function of nanoparticle concentration. The drainage rate decreased by a factor of about 50 after 50 mL of liquid was separated from SC-CO_2_ foam, and this phenomenon reveals that the adsorption of NPs on bubble lamellae occurred very slowly. In the initial stage of the foam formation, NPs mainly remained in the liquid phase and the drainage rate was very high, thus generating an unstable foam film (Figure 4A). With the prolonged aging time, NPs tended to rest at the liquid-gas interface (Figure 4B). In addition, the solution color of foaming agents was gray before the foaming test (Figure 5A). However, after the foaming test (Figure 5B), the solution color became nearly clear (Figure 5C), which indicated that NPs were adsorbed at the liquid-gas interface rather than in the aqueous phase. The adsorption of NPs at the gas-liquid interface has an effect on the disjoining pressure, which includes electrostatic, steric, and structural interactions (Exerowa et al., 1987). As the film thickness decreased further due to the film drainage by gravity, the disjoining pressure increased to a maximum at a critical film thickness. If the van der Waals interactions exceeded the electrostatic repulsion, the disjoining pressure would decline sharply (Figure 4A). Hence, the film became very unstable and large drainage rate was obtained. However, with continuous adsorption of NPs at the gas-liquid interface, a large repulsive steric forces was induced, which could raise the disjoining pressure (Figure 4B). With the increase in disjoining pressure, foam became stable and a slow drainage rate was attained (Dhanuka et al., 2006).

**Table 2:** The initial foaming volume and drainage rate as a function of nanoparticle concentration.

**Foaming agents**	**Initial foaming volume/mL**	**V_**drainage**_ < 50 mL**	**V_**drainage**_ > 50 mL**
		**Drainage rate** **(mL/min)**	**Drainage rate** **(mL/min)**
0.05% HHSB	570	81.08	52
0.05% HHSB + 0.3% NPs	545	2.84	0.055
0.05% HHSB + 0.5% NPs	535	1.76	0.048
0.05% HHSB + 0.7% NPs	520	1.48	0.029

### Effect of Salinities and Temperature on SC-CO_2_ Foam

**Figure 6:**
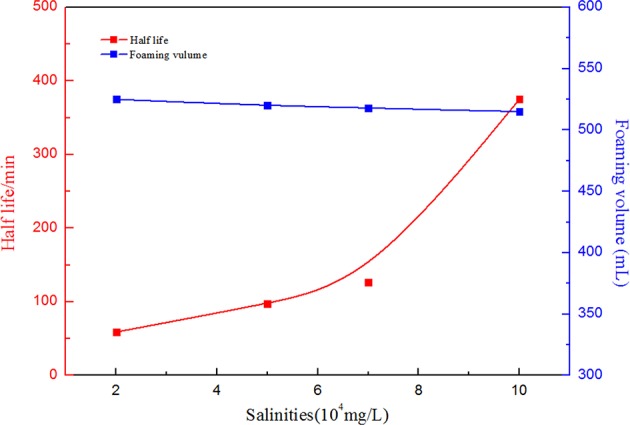
Foaming volume and half-life as a function of salinities at 70°C and 8 MPa. for SC-CO_2_ foam stabilized by 0.05% HHSB and 0.5% NPs.

**Figure 7:**
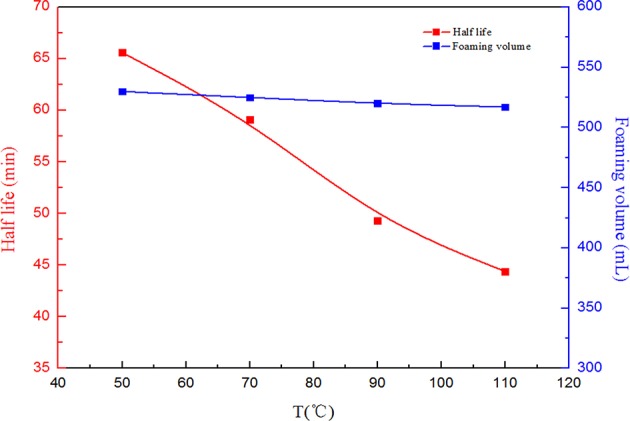
Foaming volume and half-life as a function of temperature at pressure of 8 MPa for SC-CO_2_ foam stabilized by 0.05% HHSB and 0.5% NPs.

When CO_2_ foam is applied for mobility control in oil fields, temperature and salinity manifest great effects on foam capacity and stability. Therefore, in order to investigate the effects of salinity and temperature on the SC-CO_2_ foam stabilized by NPs, the half-life of the foam was used to characterize its stability. Figure 6 depicts the effects of salinity on the foaming volume and the half-life of SC-CO_2_ foam. It is noticeable that with an increase in salinity, the foaming volume decreased slightly and the half-life of the foam increased greatly. This is attributed to the continuous adsorption of NPs on the gas-liquid interface. The dynamic light scattering test revealed that the mean zeta potential of NPs was about −22.63 mV. In the presence of salt ions, negative charges at nanoparticles surface were screened and the electrostatic repulsion between NPs became weak, thus accumulating more NPs on the gas-liquid interface to enhance the stability of SC-CO_2_ foam (Basheva et al., 1999; Adamczyk, 2003; Gupta and Basu, 2005). Figure 7 illustrates the changes in foaming volume and half-life of SC-CO_2_ foam at different temperatures. It is evident that with the increasing temperature, the drainage rate increased rapidly, thus resulting in the destabilization of SC-CO_2_ foam. Although increasing temperature made nanoparticle stability decline and facilitated the adsorption of nanoparticles at gas-liquid interface, the gas transfer rate across the film was accelerated at high temperatures, thereby causing film thinning and bubble rupture.

### Apparent Viscosity of SC-CO_2_ Foam

**Figure 8:**
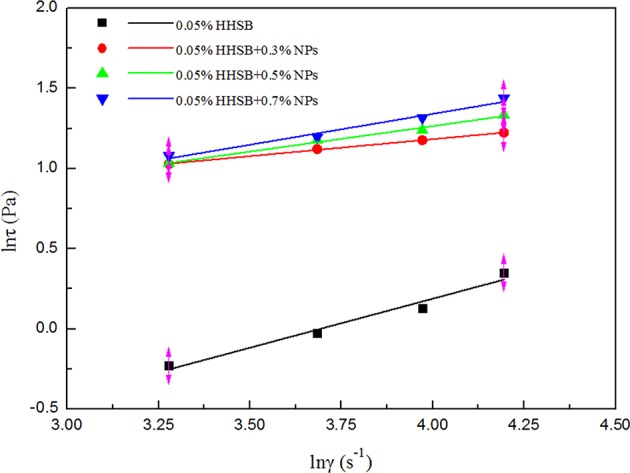
Fit curves between shear rate and apparent wall shear stress for SC-CO_2_ foams with different nanoparticles.

**Figure 9:**
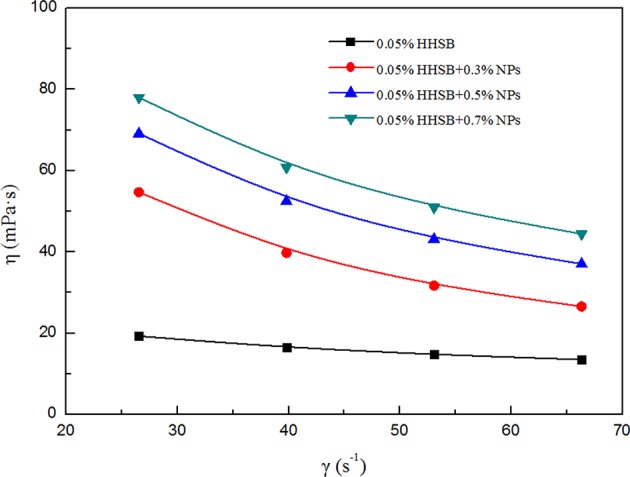
Apparent viscosity of SC-CO_2_ foam as a function of shear rate with different nanoparticles at 70°C and 8 MPa.

The power-law model was adopted to describe the non-Newtonian behavior of SC-CO_2_ foam. The logarithmic curves of shear stress vs. shear rate and the corresponding fitted curves are displayed in Figure 8. Table 3 depicts the necessary parameters and equations of the power-law model. Slopes less than unity indicates pseudoplastic fluids, whereas slopes greater than unity imply dilatant fluids. Based on the power-law model, the apparent viscosity of SC-CO_2_ foam was calculated as a function of shear rate. Figure 9 presents the apparent viscosity of the NPs-stabilized SC-CO_2_ foam at 70°C and 8 MPa. It is discernible that SC-CO_2_ foam behaved in shear-thinning or a pseudo-plastic manner and its apparent viscosity started to decline with the increasing shear rate. It is evident that NP concentration significantly affected the rheological behaviors of SC-CO_2_ foam. SC-CO_2_ foam without NPs had a flow behavior index *n* of 0.61, whereas the flow behavior index decreased to 0.2~0.4 for the NPs-stabilized CO_2_ foam. Furthermore, SC-CO_2_ foam without NPs exhibited relatively low apparent viscosity (the values were <20 mPa·s at different shear rates). With an increase in NP concentration, the apparent viscosity of SC-CO_2_ foam increased greatly. When NP concentration was 0.7%, the apparent viscosity reached 80 mPa·s at a shear rate of 26 s^−1^. It happened because the apparent viscosity of SC-CO_2_ foam was controlled by the foam structure and lamella properties (Bonilla and Shah, 2000). SC-CO_2_ foam was generated by coinjecting foaming agents and SC-CO_2_ through 50 μm pores of the foam generator and then flowing through the capillary tube. The initial bubble size was the same for SC-CO_2_ foams stabilized by different foam agents. However, the foam structure started to coarsen as foam flowed in the capillary tube due to gas transfer and capillary pressure. At the same shear rate, more NP adsorption at the liquid-gas interface would provide thick protective shells around the bubbles against foam coarsening and gas transfer, thereby resulting in dense texture and higher apparent viscosity (Alargova et al., 2004; Stephanie et al., 2008).

**Table 3:** The parameters of power-law equations for the SC-CO_2_ foam with different NPs.

**Nanoparticle concentration/%**	***n***	***K*'**	***K***	**Equation**
0	0.61	0.080	0.073	τ = 0.073γ^−0.39^
0.3	0.21	1.40	1.22	τ = 1.22γ^−0.79^
0.5	0.32	0.99	0.86	τ = 0.86γ^−0.68^
0.7	0.39	0.82	0.72	τ = 0.72γ^−0.61^

### Evaluation of Resistance Factor During the Core Flooding Experiment

**Figure 10:**
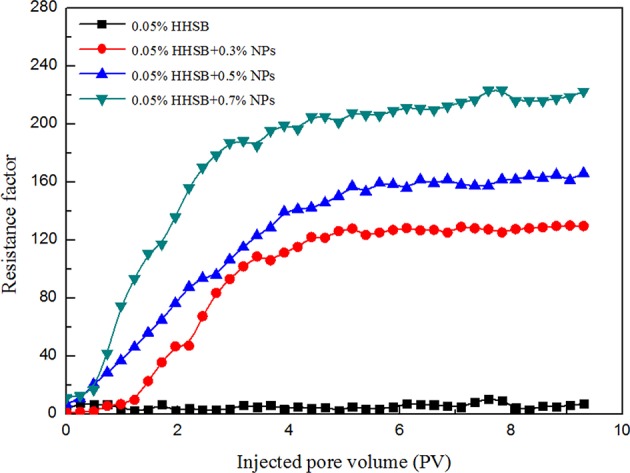
The steady-state resistance factors of SC-CO_2_ foam in the core flooding as a function of injected pore volume at 70°C and 8 Mpa.

Resistance factor is used to characterize the strength of the SC-CO_2_ foam. A large resistance factor means lower foam mobility, which depends considerably on foam texture. The effects of NP concentration on resistance factor are presented in Figure 10. It is noticeable that the value of the resistance factor gradually increased with the increasing NP concentration. It could be deduced that the NPs-stabilized SC-CO_2_ foam had much finer foam texture and denser bubble distribution. The gravitational drainage of liquid through foam lamellae was not the main mechanism of foam rupture in the porous medium. When the foam flowed through the porous medium, the main mechanisms of foam rupture were lamellae collapse due to capillary pressure and gas diffusion across foam lamellae. It can be inferred that NPs significantly enhanced the stability of SC-CO_2_ foam in the porous media by reducing bubble coalescence rates. In order to explore the mechanisms of increased resistance factor in the presence of NPs in the porous media, the stochastic population balance (SPB) model was employed to describe the flow behavior of the NPs-stabilized SC-CO_2_ foam (Zitha and Du, 2010; Du et al., 2011). In the SBP model, the bubble generation in the porous media was regarded as a macroscopic stochastic process. The net foam generation (*q*_*f*_) was obtained by Equation (9).

(10) $$\begin{array}{r} q_{f}=\varphi S_{f}K_{g}\left(n_{\infty }-n\right) \end{array}$$

where φ is the porosity of the core, *S*_*f*_denotes foam saturation, *K*_*g*_is the foam generation rate, n_∞_ is the maximum bubble density, and *n* signifies bubble density.

**Figure 11:**
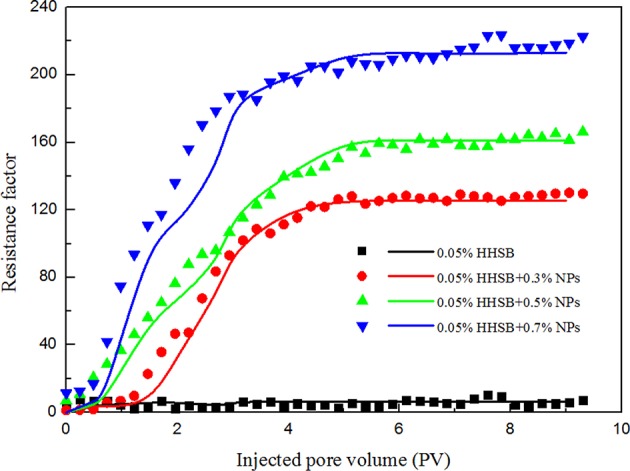
Experimental and simulation results for SC-CO_2_ foam at 70°C and 8 Mpa (Dots represent experimental results and lines represent simulation results).

With involving two parameters of foam generation rate *K*_*g*_ and maximum bubble density *n*_∞_, the SBP model shows considerable conveniences to characterize the strength of NPs-stabilized SC-CO_2_ foam. Bubble density was controlled by bubble generation and coalescence mechanisms: capillary snap-off, lamella division and leave behind generated foams and increased the bubble density, whereas gas transfer and capillary suction destroyed foams and diminished the bubble density. With addition of nanoparticles, the gas transfer rate between bubbles was decreased and the ability to withstand capillary pressure was improved in porous media, thereby resulting in larger maximum bubble density and higher generation rate. The control equations of the two-phase flow process in the one-dimensional domain were solved by the IMPES algorithm. The simulation parameters of the SBP model are presented in Tables S1–S4. Figure 11 illustrates the obtained experimental and simulation results for the SPB model, and it is showed that simulation and experimental results were in good agreement for the resistance factor.

### Microstructures of SC-CO_2_ Foam

**Figure 12:**
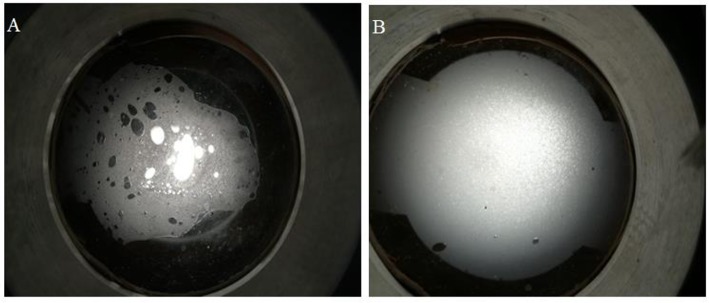
Digital photographs of SC-CO_2_ foams stabilized by 0.05% HHSB surfactant **(A)** and 0.05%+0.5% NPs **(B)** at 70°C and 8 MPa.

**Figure 13:**
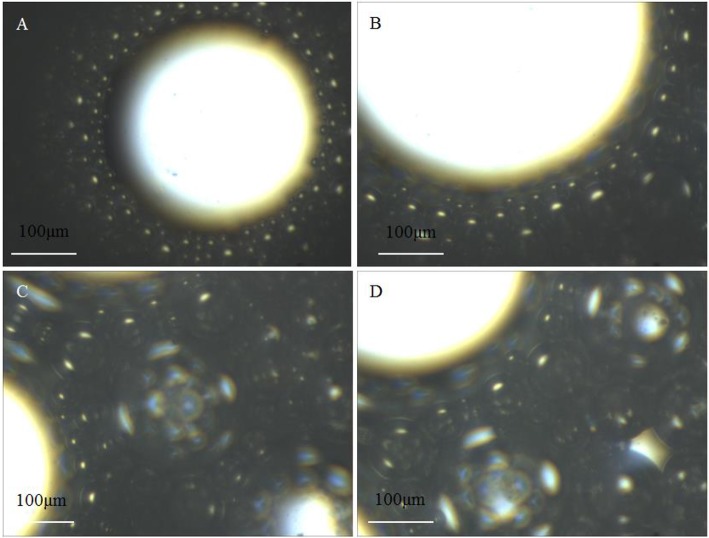
Micrographas of SC-CO_2_ foams stabilized with 0.05 and 0.5% NPs HHSB at 70°C and 8 MPa aging for 10 min **(A)**, 40 min **(B)**, 70 min **(C)**, and 100 min **(D)**.

The microstructure of SC-CO_2_ foam from the exit of the core was observed by the microscope. Figure 12 displays the digital photographs of SC-CO_2_ foams with and without NPs immediately after foam flow was stopped. SC-CO_2_ foam with NPs had a more compact bubble structure, which blocked nearly all the light below the optical cell. In order to distinguish SC-CO_2_ foam texture, Figure 13 displays the microscope images of SC-CO_2_ foams as a function of time. The mean bubble size was about 20~30 μm after the foam flowed into the high-pressure cell for 10 min. Since the space between sapphire windows was about 100 μm, multiple bubble layers existed in the cell. With the prolonged experiment, the mean bubble size increased for SC-CO_2_ foams with and without NPs. However, the increase rate of NPs-stabilized SC-CO_2_ foams was lower than that of the foams without NPs. After executing aging for 1 h, the mean bubble size of NPs-stabilized SC-CO_2_ foams increased to 40~50 μm, whereas the mean bubble size of the foams without NPs reached 70~80 μm. It can be concluded that coalescence rate of SC-CO_2_ foam became slower with increasing nanoparticle concentration.

### Surface Elasticity of SC-CO_2_ Foam

**Figure 14:**
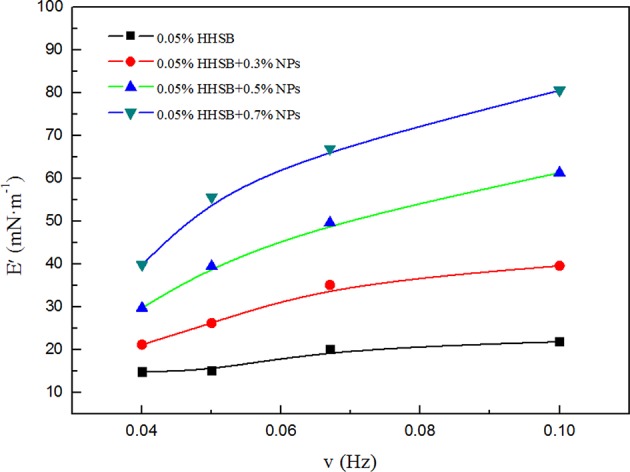
Dilatational surface elasticities E as a function of frequency for SC-CO_2_ foams with different nanoparticles at 70°C and 8 MPa.

The foam stability was related to the surface interfacial rheological properties of the bubble film. A large dilatational surface elasticity could dampen external film fluctuations induced by temperature, pressure, or salinity (Heller and Kuntamukkula, 1987; Basheva et al., 1999; Adkins et al., 2010). In order to explore the mechanisms of foam stabilization, the dilatational surface elasticity between CO_2_ and foam agents were measured as a function of frequency at 70°C and 8 MPa. It is noticeable that the dilatational surface elasticity was increased with increasing NP concentration (Figure 14). When NP concentration reached 0.7%, the dilatational surface elasticity could reach up to 4 times as large as that of surfactant solutions without NPs. Surfactant adsorption was a dynamic process where adsorption and desorption are present simultaneously, and surfactant molecules adsorbed at the CO_2_-water interface easily underwent desorption from the interface due to external disturbance (Li et al., 2017). In comparison to HHSB molecules, NPs were adsorbed more strongly at the CO_2_-water interface, thus it was difficult for them to be desorbed from the interface. The energy of attachment (E), which is required to remove NPs from the solid-liquid interface, can be expressed as (Stephanie et al., 2008).

(11) $$\begin{array}{r} E=\pi R^{2}\gamma_{\text{CW}}{\left(1\pm \cos\theta \right)}^{2} \end{array}$$

where *r* is the particle radius, γ_*CW*_ is the interfacial tension between SC-CO_2_ and water, θ is the contact angle particles make with the water phase.

**Figure 15:**
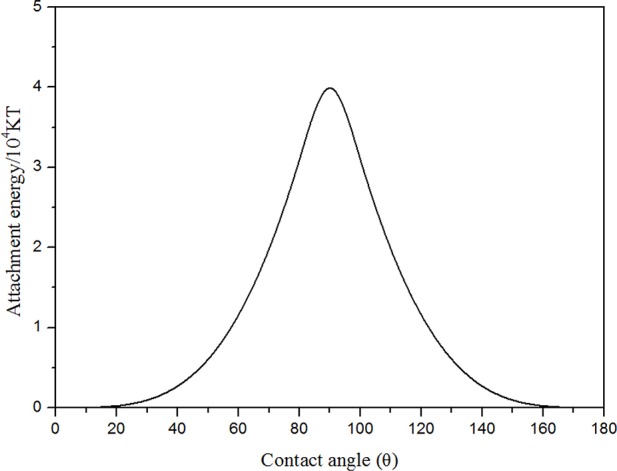
The energy of attachment E′ for NPs at the interface as a function of contact angle θ at 25°C (*R* = 65 nm, γ_cw_ = 16 mN·m^−1^).

For hydrophilic NPs (0° < θ < 90° and cos θ > 0), the sign in the bracket would be negative. For hydrophobic NPs (90° < θ < 180° and cos θ < 0), the sign in the bracket would be positive. Based on the above equation, the energy of attachment was calculated as a function of θ, and the corresponding results are displayed in Figure 15. It is noticeable that NPs were most strongly adsorbed at the interface for 85° < θ < 95°. The hydrophilic nanoparticles were dispersed in water phase and it can be inferred that the contact angle particles make with the water phase is lower than 90°. If the contact angle was >20°, the energy of attachment was large on the order of magnitude of 10^4^ KT. Therefore, when the bubble film confronted external disturbances (temperature and capillary pressure), NPs were strongly adsorbed at the CO_2_-water interface due to the large energy of attachment. NPs adsorbed at the CO_2_-water interface could result in higher dilatational surface elasticity, which dampened external disturbances and resisted the deformation of foam lamellae (Basheva et al., 1999; Stubenrauch and Miller, 2004).

## Conclusions

A novel foaming agent composed of zwitterionic surfactant and silica nanoparticles was developed to stabilize SC-CO_2_ foam. The foaming ability and stability of SC-CO_2_ foam was investigated at 70°C and 8 MPa. The drainage rate of the SC-CO_2_ foam first was fast and then started to decelerate with the increase in NP adsorption at the gas-liquid interface. NP adsorption at the interface could enhance the disjoining pressure, thereby preventing film thinning. The stability of SC-CO_2_ foam got improved with the increase in salinity due to the screened electrostatic repulsion between negatively-charged silica NPs. NP-stabilized SC-CO_2_ foam showed power-law shear thinning rheological behavior and the apparent viscosity increased up to 80 mPa·s with increasing nanoparticle. The texture of SC-CO_2_ foam was observed during steady-state foam flow, and the average effluent bubble size was found as 10~20 μm. The coalescence rate of SC-CO_2_ foam gradually became very slow with the increasing NP concentration. Based on the SBP model, the resistance factor of SC-CO_2_ foam was simulated as a function of NP concentration by considering its generation rate and maximum bubble density. The resistance factor obtained from the SBP model agreed well with experimental results. Oscillation measurements proved the improvement of SC-CO_2_ foam stability with the increasing NP concentration was attributed to an increase in surface elasticity.

## Data Availability Statement

The datasets generated for this study are available on request to the corresponding author.

## Author Contributions

WL participated in the design of the manuscript and drafted the manuscript. FW, CX, and JO came up with ideas for the manuscript. LS analyzed experimental results. MD carried out experiments. DD and PL revised the manuscript.

### Conflict of Interest

The authors declare that the research was conducted in the absence of any commercial or financial relationships that could be construed as a potential conflict of interest.
