# Supplementary material for: A Novel Supercritical CO2 Foam System Stabilized With a Mixture of Zwitterionic Surfactant and Silica Nanoparticles for Enhanced Oil Recovery
Source: Front Chem. 2019 Oct 29;7:718. doi: 10.3389/fchem.2019.00718 (PMC6828982; doi:10.3389/fchem.2019.00718)
Supplement: Supplementary file 1 [file Table_1.DOCX]

Supporting Information

A Novel Supercritical CO_2_ Foam System Stabilized with a Mixture of Zwitterionic Surfactant and Silica Nanoparticles for Enhanced Oil Recovery

*Weitao Li* ^^[[1]](#footnote-1)^*^*^a^, Falin Wei ^a^, Chunming Xiong ^a^, Jian Ouyang ^a^, Liming Shao ^a^, Mingli Dai ^a^, Dongxing Du ^b^, Pingde Liu ^a^*

^a^ Research Institute of Petroleum Exploration & Development, PetroChina, Beijing, 100083, P. R.China

^b^ College of Electromechanical Engineering, Qingdao University of Science and Technology, Qingdao, 266061, P. R.China

**Table S1** The parameters employed in the SPB model for SC-CO_2_ foam stabilized with 0.05% HHSB.

| PV | 0.5 | 0.7 | 1.5 | 2 | 3 | 4 | 5 | 6 | 7 | 10 |
| --- | --- | --- | --- | --- | --- | --- | --- | --- | --- | --- |
| k_g_ | 1 | 1 | 1 | 0.5 | 0.1 | 0.01 | 0.01 | 0.01 | 0.01 | 0.01 |
| n_max_ | 30 | 30 | 30 | 30 | 30 | 30 | 30 | 30 | 30 | 30 |

**Table S2** The parameters employed in the SPB model for SC-CO_2_ foam stabilized with 0.05% HHSB and 0.3% NPs.

| PV | 0.5 | 0.7 | 1.5 | 2 | 3 | 4 | 5 | 6 | 7 | 10 |
| --- | --- | --- | --- | --- | --- | --- | --- | --- | --- | --- |
| k_g_ | 0.001 | 0.001 | 0.0005 | 0.0001 | 0.01 | 0.05 | 0.05 | 0.1 | 0.1 | 0.1 |
| n_max_ | 1000 | 1000 | 1000 | 1000 | 1000 | 1000 | 1000 | 1000 | 1000 | 1000 |

**Table S3** The parameters employed in the SPB model for SC-CO_2_ foam stabilized with 0.05% HHSB and 0.5% NPs.

| PV | 0.5 | 0.7 | 1.5 | 2 | 3 | 4 | 5 | 6 | 7 | 10 |
| --- | --- | --- | --- | --- | --- | --- | --- | --- | --- | --- |
| k_g_ | 0.001 | 0.001 | 0.0005 | 0.001 | 0.05 | 0.05 | 0.05 | 0.05 | 0.1 | 0.1 |
| n_max_ | 1000 | 1000 | 1000 | 1000 | 1000 | 1000 | 1000 | 1000 | 1000 | 1000 |

**Table S4** The parameters employed in the SPB model for SC-CO_2_ foam stabilized with 0.05% HHSB and 0.5% NPs.

| PV | 0.5 | 0.7 | 1.5 | 2 | 3 | 4 | 5 | 6 | 7 | 10 |
| --- | --- | --- | --- | --- | --- | --- | --- | --- | --- | --- |
| k_g_ | 0.01 | 0.01 | 0.001 | 0.001 | 1 | 10 | 10 | 0.1 | 0.1 | 0.1 |
| n_max_ | 1000 | 1000 | 1000 | 1000 | 1000 | 1000 | 1000 | 1000 | 1000 | 1000 |

1. *Corresponding authors

   *E-mail addresses:* [liweitao@petrochina.com.cn](mailto:liweitao@petrochina.com.cn), Tel: +86-010-83592346 Fax: +86-010-83592346 [↑](#footnote-ref-1)
